# Impact of Hemoglobin Levels During Definite Chemoradiotherapy of Patients with Locally Advanced Head and Neck Squamous Cell Carcinoma on Survival

**DOI:** 10.3390/medicina61112027

**Published:** 2025-11-13

**Authors:** Sandy Hazko, Amed Ahmed, Robert Michael Hermann, Mathias Alexander Sonnhoff, Athanasia Warnecke, Frank Bruns, Robert Blach, Hans Christiansen, Jan-Niklas Becker

**Affiliations:** 1 Department of Radiotherapy, Hannover Medical School, 30625 Hannover, Germany; 2 Department of Otorhinolaryngology-Head & Neck Surgery, Hannover Medical School, 30625 Hannover, Germany; 3 Centre for Radiotherapy and Radiooncology, 26655 Westerstede, Germany

**Keywords:** hemoglobin, anemia, head and neck cancer, chemotherapy, radiotherapy

## Abstract

*Background and Objectives*: This study aims to investigate the impact of hemoglobin (Hb) level changes during radiochemotherapy (RCT) on the survival of patients with locally advanced head and neck squamous cell carcinoma (HNSCC). *Materials and Methods*: A retrospective analysis was conducted on 97 patients with HNSCC, treated with definitive RCT between January 2016 and October 2021. Hb levels were monitored weekly during RCT. Kaplan–Meier and Cox regression analysis were performed. *Results*: There was a significant association between Hb levels at the end of RCT and overall survival (*p* < 0.01). Initial Hb levels and Hb level changes were not significantly associated with survival. In multivariate analysis, a lower body mass index (BMI) and Hb levels at week six were identified as significant prognostic factors. *Conclusions*: At the end of RCT, rather than baseline levels or changes during treatment, Hb levels are a significant prognostic factor for overall survival in patients with HNSCC.

## 1. Introduction

The primary treatment of squamous cell carcinoma in the head and neck area (HNSCC) usually consists of surgical resection, if the tumor is local and non-advanced, with reconstruction of the defect. Advanced or inoperable HNSCC are treated curatively with radiotherapy in combination with chemotherapy (RCT) [1,2]. However, many patients have a low survival probability or show local progression despite the established RCT [1]. A possible reason for the failure of RCT is tumor hypoxia in addition to radioresistance [3]. The radiobiological mechanism of action of external beam irradiation (RT) used in HNSCC patients is the induction of double-strand breaks within the DNA. Oxygen enhances this effect through the process of oxygen fixation, which stabilizes radiation-induced DNA damage and prevents its repair. Therefore, a higher oxygen concentration provided by a higher amount of red blood cells is expected to be a major key in sufficient tumor cell damage by RT. Polarographic oxygen measurement studies showing the relationship between hemoglobin (Hb) concentration, tumor control, and survival rates support this assumption. In addition, many studies have shown that 40% of HNSCC patients develop anemia during RCT [4,5].

On the other hand, aggressive tumor activity is postulated to be a major cause of anemia [6]. A low Hb level before RCT is associated with poorer treatment outcomes and lower survival rates. Studies have shown the influence of Hb levels before the start or at the end of RCT on overall and progression-free survival [7,8,9,10,11,12]. Often, Hb level changes during the six to eight weeks of RCT [13]. This is postulated to be due to a depression of bone marrow stem cells by the simultaneously given chemotherapy agents or through malnutrition because of RCT-induced mucositis resulting in dysphagia with lower amounts of crucial nutrients needed to produce red blood cells [14]. Therefore, besides the initial red blood cell counts, the change in Hb levels during RCT may have an impact on the oncological outcome. Whereas the prognostic role of static Hb levels before or after treatment is established, no prior study systematically correlated dynamic weekly Hb trajectories throughout RCT with patient survival.

Consequently, this study aims to analyze the impact of Hb level changes during RCT on the outcome of HNSCC patients.

## 2. Materials and Methods

The results of this study relate to patients with histologically confirmed HNSCC with tumor sites of the pharynx (naso-, oro-, and hypopharynx), larynx, and oral cavity. Only patients without any metastases and without prior resection receiving RCT with curative intent between January 2016 and October 2021 were included, and the results of this study were consecutively included and analyzed. Patients not completing the RCT due to incompliance or disease progression were excluded. Th study has been conducted in full accordance with ethical principles, including the World Medical Association Declaration of Helsinki (version 2002). The Medical Ethics Research Committee of Hannover Medical School approved the study’s design. Patient consent was waived due to the retrospective nature of this study.

Overall, RT was performed using a hypofractionated volumetric modulated arc therapy with simultaneous integrated boosts of 30 fractions (5 per week). Elective cervical nodes received a total dose of 54 Gy (dose per fraction 1.8 Gy) and morphological infiltrated lymph nodes 60 Gy (dose per fraction 2.0 Gy). The gross tumor volume was boosted to a total dose of 66 Gy (dose per fraction 2.2 Gy) [15,16]. At the same time, the patients received weekly chemotherapy with cisplatin or carboplatin/paclitaxel intravenously for six weeks of treatment. The cisplatin dosage was 40 mg per square meter of the body surface, accumulating to 240 mg. Patients with contraindications to cisplatin (mainly due to reduced renal function) received weekly carboplatin (AUC2) and paclitaxel (45 mg per m2 body surface area). In cases of infections with fever or white blood cell count below 3.0 × 10^9^/L and/or platelets below 75 × 10^9^/L, chemotherapy was postponed till those conditions had improved. Analyses were performed only for patients who were not transfused with red blood cell concentrates or erythropoietin to determine the influence of Hb levels and preserve the biological information of spontaneous Hb variation.

From the beginning until the end of RCT, weekly blood samples with a blood count and serum creatinine concentrations were recorded and evaluated once a week for six weeks. Furthermore, information on chemotherapy, including the complications mentioned above to postpone a cycle, was noted weekly. Height and weight were also measured prior to RCT. Additionally, toxicities of the skin, mucosa, and feeding (dysphagia), including the form of feeding, were categorized weekly based on the Common Terminology Criteria for Adverse Events (CTCAE) v.5.0 [16]. Supportive care measures (nutritional counseling, speech therapy, PEG placement) followed institutional standards. The characteristics of the patients examined in this study also include the TNM grading according to AJCC staging [17], gender, age at diagnosis, and other risk factors such as smoking and alcohol consumption. Patients were called in for follow-up every 3 months for 2 years after RCT. Thereafter, they were seen once a year for five more years, completing the follow-up.

Descriptive statistics of the patient cohort stratified by the mean Hb level were performed for weeks 1 (treatment beginning) and 6 (end of treatment), as well as the mean difference between week 1 and week 6. Continuous data were analyzed for normal distribution using the Kolmogorov–Smirnov test with additional Q-Q plots and then given with the standard deviation. Categorical data were given as a number with respective percentages. Patient characteristics were compared with a chi-square test for categorical data and an independent *t*-test for continuous data. Survival analysis with Kaplan–Meier was carried out for the Hb levels of week 1 and week 6, and the decrease between week 1 and week 6 was stratified by the respective means and analyzed using the corresponding log-rank. Overall survival was defined as the time from the start of RCT to death and was calculated in months. Progression-free survival was defined as time to recurrence or disease progression such as metastasis or secondary tumor or death. Patients who were alive or progression-free were censored at their last clinical contact. Furthermore, a cox-regression univariate and multivariate analysis was performed to determine the hazard ratio of the Hb levels and other factors on survival. A *p*-value below 0.05 was considered statistically significant within this analysis, performed with IBM Statistical Package for Social Sciences (SPSS) (IBM, Somers, NY, USA) version 27.

## 3. Results

Ninety-seven patients met the inclusion criteria. The average age was 63 years old, ranging from ages 43 to 79. The tumor stage was classified according to the American Joint Committee of Cancer (AJCC) [17], and patients were divided into groups accordingly. Twelve patients had tumor stage II, 20 patients had stage III, and 65 patients had stage IV (Table 1).

Hb levels at the start of RCT ranged from 8.1 to 15 g/dL, with a mean value of 12.6 g/dL and a standard deviation of 1.74 g/dL. No patient received a red blood cell concentrate transfusion during the six weeks of RCT. At the end of RCT, Hb values were between 8.2 and 13.6 g/dL, with a mean value of 10.31 g/dL and a deviation of 1.57 g/dL. Eighty-two patients had Hb levels below 12 g/dL according to the WHO definition of anemia during treatment (15). There was a significant difference between men and women (*p* = 0.04) for the first week. In week six, the differences were less pronounced; between weeks one and six, they were not significant. There was no significant difference between the age of the patients and the decrease in Hb levels. Risk factors such as tobacco consumption and alcohol in the patients, whose Hb level was below and above the mean value, showed no significant influence according to the Chi-Square test.

Regarding height, there was no significant effect on the change in Hb levels, while weight showed a significant difference. Consequently, the body-mass index had a significant effect on the Hb drop. Furthermore, tumor location, T and N classification, tumor stage, chemotherapy (cisplatin vs. paclitaxel with carboplatin), and RT-associated dysphagia showed no significant difference and influence on Hb decrease during the six weeks of RCT. There were no missing data.

**Figure 1:**
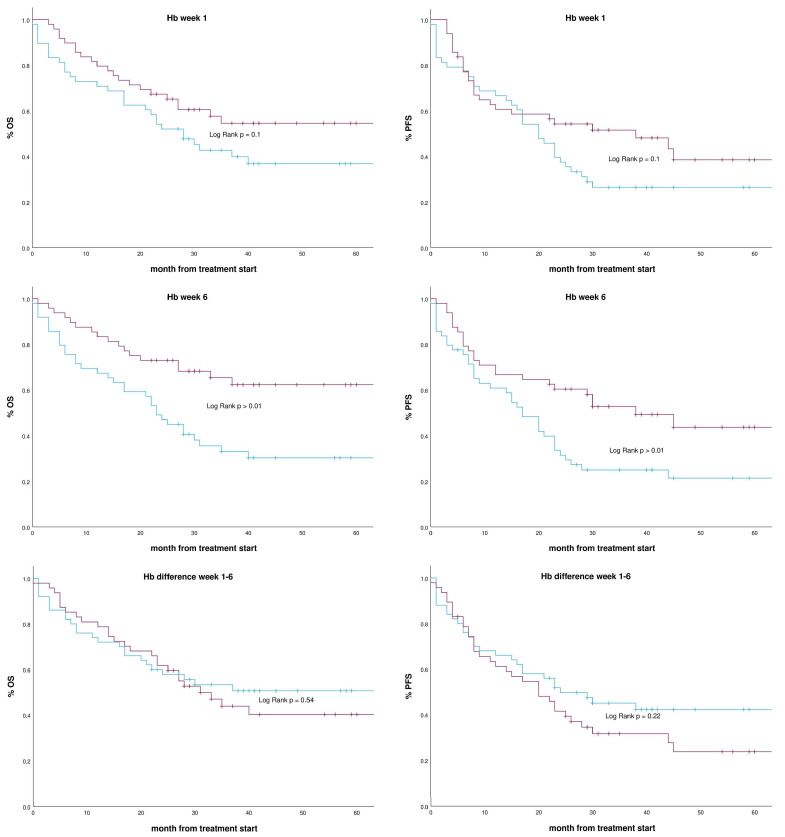
Kaplan–Meier survival analysis with respective Hb stratification. Red line above Hb mean (week 1 12.6 g/dL (49 of 97 patients), week 6 10.3 g/dL (48 of 97 patients), Hb difference week 1–6 2.29 g/dL (47 of 97 patients)) blue line equal or below mean, + censored; OS overall survival, PFS progression-free survival; Significance *p* for the respective Log-rank test.

The mean Hb value of the first week of RCT was 12.6 g/dL, stratifying patients equal to or below the mean and above the mean. The Kaplan–Meier analysis in Figure 1 shows no significant influence on overall survival (log-rank test *p* = 0.1). In contrast, a stratification by the mean Hb value of the sixth week of RCT showed a significant influence (log-rank test *p* > 0.01). The mean Hb value of week six of RCT dropped to 10.3 g/dL with a mean difference from week one to six of 1.57 g/dL. Stratifying patients using the Hb difference in week one to week six, the log-rank test gave no significant result (*p* = 0.54). Similar results were obtained in the respective progression-free survival analysis. Th median follow-up time was 28 months.

Cox regression was performed as stated in Table 2. The levels of Hb from week one to week six were analyzed with an increasing hazard ratio towards week six and a corresponding decreasing *p*-value. In the univariate analysis, the BMI was significant, whereas gender, age, and Hb level of the first week, as well as the Hb difference from week one to week six, are above a *p* of 0.05. Within the multivariate analysis, only the BMI and Hb levels of week six, which were the most significant Hb levels during RCT, were included. Weeks two to five were excluded due to their dependence on each other. Both BMI and Hb levels of week six were also significant in multivariate Cox regression analysis.

## 4. Discussion

This study identified the Hb level at the end of RCT as a significant prognostic factor for overall survival. The initial Hb level was less significant, as was the progression of anemia during RCT.

Research has already found the effect of pretreatment Hb levels as an independent prognostic factor for outcomes after RCT in HNSCC patients [18,19]. To our knowledge, the prognostic value of Hb levels at the end of RCT was only analyzed for (nasopharynx) and larynx carcinoma in previous studies [7,8,9,10,11,12].

In our study population, we observed a decrease in the mean Hb from 12.6 g/dL to 10.3 g/dL. Chemotherapy affects the hemopoietic cells, thus lowering the number of newly produced red blood cells. On the other hand, progressing dysphagia due to RT toxicity on the mucosa of the upper digestive tract can worsen the hemopoiesis. Due to both factors, hemoglobin levels often decrease during treatment [12].

In comparison with the pretreatment levels of Melo-Alvim et al. [12], more of our patients, roughly 46%, showed a Hb below 12.6 g/dL in the first week (comparable to a pretreatment value) in comparison to about 36% below 12.5 g/dL. In their study, with a younger population (58 years vs. 63 years in our population), the pretreatment of Hb stratification by 12.5 g/dL also showed significant effects on survival. Observing the Kaplan–Meier curves survival is somewhat comparable to our study, with about 50% of the patients living after 60 months in the equal or above Hb of 12.5 g/dL group and roughly 30% in the group below 12.5 g/dL Hb—with slightly more survivors in our study (about 35% after 60 months). The same is valid for progression-free survival. Melo-Alvim et al. made a stratification using the literature without stating any mean Hb values of their populations, which could explain the relatively small difference. To our knowledge, no direct survival data on Hb values at the end of the RCT or after RCT is available, as published studies hinder a direct comparison.

The trend of decreasing hazard ratios towards the end of RCT from week to week, with the most significant values in the last week, is another major aspect of this study. No other studies have shown this effect before. An explanation could be the effect of a tumor response. Reduction in tumor activity could lead to less inflammatory effects and, thus, less destruction of red blood cells. Malignant tumors can cause an increase in inflammatory immune effects, including the upregulation of different cytokines. These decrease the life span of erythrocytes and hamper reproduction through lower iron metabolism. Thus, low end-of-treatment Hb levels appear to mirror residual tumor burden, supporting their interpretation as an associative disease marker rather than a causal modifier of radiotherapy response [20].

A randomized controlled trial has shown that the correction of Hb levels through transfusion [4] does not improve outcomes in patients with low Hb concentrations. The same was found for the use of erythropoietin. These findings would contribute to the hypothesis that Hb concentration is more a prognostic disease marker due to tumor activity than an indicator of impaired therapeutic efficacy due to lower oxygenation [21].

Furthermore, patients with a Hb count below the mean in week six had significantly higher toxicity in comparison to week one. This may be explained by less nutrient intake with a higher degree of dysphagia, which might decrease red blood cell generation, as shown in previous studies [22]. Conversely, this may influence the body composition, and the tumor immune response may also affect tumor control [23]. Here, sufficient nutritional support, including gastro tube or parenteral feeding, may be necessary to compensate for oral nutrient loss [24].

Hb differences between the first and last week of treatment did not significantly affect survival. This may be because of chemotoxicity in the bone marrow. Patients with more affection of the bone marrow due to chemotherapy show a more significant difference between the beginning of RCT (first week) and the end (sixth week), with the steady accumulation of the weekly chemotherapy toxicity. This, on the other hand, is not a prognostic factor on overall or progression-free survival. The same is seen when stratifying using the Hb difference between weeks one and six, where all tumor sites showed similar percentages regardless of tumor T status.

Separated by mean, Hb levels were generally significantly associated with sex and weight, which can be related to a higher red blood cell count in men and, to some extent, positively correlated with body weight [25]. In the Cox regression, sex was not a significant factor for overall survival, whereas body mass index was a major prognosis factor in multivariate regression survival analysis. An explanation could be the negative effect of a low BMI on survival, as elaborated by different studies [26,27]. Here, also, the effect of dysphagia through the tumor itself as part of the RCT-associated toxicity could negatively impact the weight and thus influence tumor control and survival in general. Compared to the Hb transfusion without a benefit, a sufficient nutritional substitution with a high caloric oral or gastral tube, or parenteral feeding, enhances the whole-body function through adequate nutrition [28]. As the connection between BMI, nutrition, and treatment tolerance is well established, prospective validation of structured nutritional support and its impact on therapy outcomes should be pursued.

In our cohort, serial Hb measurements demonstrated prognostic relevance, particularly toward the end of RCT. Building on this finding, future work could refine Hb-based risk assessment by integrating additional laboratory markers such as albumin and globulin into predictive models. Similar nomograms, combining TNM stage and hemoglobin–albumin–globulin ratio, have already proven prognostic value for overall and progression-free survival in nasopharyngeal carcinoma [29].

Hemoglobin could be more than just a predictive marker in patients treated with RCT. In patients with recurrent or metastatic head and neck squamous cell carcinoma that were treated with cetuximab-containing regimens, several hematological parameters have been shown to be associated with poor overall survival outcomes. These parameters include increased white blood cell count, decreased hemoglobin levels, increased platelet count, increased absolute neutrophil count, decreased absolute lymphocyte count and increased neutrophil-to-lymphocyte ratio. The identified parameters reflect alterations in both the immune system and overall hematological status, which may influence treatment efficacy and patient outcomes [29]. These results highlight the importance of comprehensive hematological assessments in HNSCC patients and suggest that these parameters could be valuable in treatment planning and prognostic evaluation [30].

The study’s strength is the homogenous treatment regime for all patients. Furthermore, blood counts were rigorously collected weekly without missing data. Limitations are that the retrospective study design may miss other relevant information, such as hematological disease information or systemic inflammation markers, for further analysis and potential adjustments. Lack of standardized nutritional and comorbidity assessment may allow residual confounding. Furthermore, different stratification Hb levels by previously published studies make comparisons challenging.

## 5. Conclusions

This is the first study showing the increasingly significant impact of low Hb levels towards the end of RCT in HNSCC. Patients with higher tumor burdens appearing less affected by RCT, potentially representing a subgroup with better prognosis and candidates for individualized follow-up schedules. Weekly Hb monitoring during RCT may help identify patients at risk, as a low end-of-treatment Hb should prompt structured nutritional and swallowing assessment. Since Hb transfusion and erythropoietin use showed no survival benefit in this population, clinical focus should rather be directed toward supportive measures such as optimizing nutrition and feeding status, which may ultimately improve outcomes. These findings should be validated in prospective, ideally interventional trials.

## Figures and Tables

**Table 1:** Characteristics with stratified hemoglobin levels.

	All Patients	Week 1		*p*	Week 6		*p*	Difference Between Week 6 and Week 1		*p*
Hemoglobin (g/dL)		≤12.6	>12.6		≤10.3	>10.3		≤1.57	>1.57	
Sex				0.04			0.05			0.78
Female	21	13 (61.90%)	8 (38.09%)		14 (66.6%)	7 (33.3%)		6 (28.5%)	15 (71.4%)	
Male	76	32 (42.10%)	44 (57.98%)		35 (46%)	41 (53.9%)		23 (30.2%)	53 (69.7%)	
Age at diagnosis (years)	63 (±8.5)	62 (±8.1)	63 (±8.8)	0.59	61 (±7.7)	64 (±9.1)	0.16	63 (±9.2)	62 (±8.2)	0.08
Smoking										
No	41	20	21	0.94	19	22	0.5	14	27	0.9
Yes	56	25	31		31	25		17	39	
Weight (kg)	74.1 (±19.2)	65.9 (±15.9)	81.2 (±19.1)	<**0.01**	69.4 (±17.1)	79 (±20.2)	**0.01**	66.3 (±15.4)	78.3 (±19.5)	<**0.01**
Length (cm)	174.3 (±7.81)	173.5 (±8.8)	175 (±6.8)	0.34	174 (±8.3)	174.7 (±7.2)	0.66	173.8 (±8.5)	174.9 (±7.3)	0.51
BMI (kg/m^2^)	24.3 (±5.8)	21.8 (±4.8)	26.4 (±5.7)	<**0.01**	22.9 (±5.2)	25.7 (±6.0)	<**0.01**	21.8 (±4.4)	25.5 (±6.0)	<**0.01**
Tumor site				0.76			0.84			0.80
Oropharynx	33 (34.02%)	17 (37.77%)	16 (30.76%)		18 (54.5%)	15 (45.4%)		12 (36.3%)	21 (63.6%)	
Larynx	8 (8.24%)	3 (6.66%)	5 (9.61%)		4 (50%)	4 (50%)		1 (12.5%)	7 (87.5%)	
Hypopharynx	19 (19.58%)	5 (11.11%)	14 (26.92%)		6 (31.5%)	13 (68.4%)		3 (15.7%)	16 (84.2%)	
Oral cavity	31 (31.95%)	18 (40%)	13 (25%)		19 (29.03%)	12 (38.7%)		12 (38.7%)	19 (29.03%)	
Nasopharynx	6 (6.18%)	2 (4.44%)	4 (7.69%)		2 (33.3%)	4 (66.6%)		3 (50%)	3 (50%)	
T-stage				0.74			0.35			1.00
T1	4 (4.12%)	1 (25%)	3 (75%)		1 (25%)	3 (75%)		1 (25%)	3 (75%)	
T2	16 (16.49%)	5 (11.11%)	11 (21.15%)		5 (31.2%)	11 (68.7%)		4 (25%)	12 (75%)	
T3	28 (28.86%)	12 (26.66%)	16 (30.76%)		17 (60.7%)	11 (39.2%)		7 (25%)	21 (75%)	
T4	49 (50.51%)	27 (60%)	22 (42.30%)		27 (55.1%)	22 (44.8%)		19 (38.77%)	30 (61.22%)	
N-Stage				0.65			0.48			0.31
N0	20 (20.61%)	11 (24.44%)	9 (17.30%)		12 (60%)	8 (40%)		4 (20%)	16 (80%)	
N+	77 (79.38%)	34 (75.55%)	43 (82.69%)		37 (48.05%)	40 (51.9%)		27 (35.06%)	50 (64.9%)	
Anatomic stage group				0.09			0.11			0.33
Stage II	12 (12.37%)	2 (4.44%)	10 (1.23%)		3 (25%)	9 (75%)		2 (16.6%)	10 (83.3%)	
Stage III	20 (20.61%)	12 (26.66%)	8 (15.38%)		12 (60%)	8 (40%)		6 (30%)	14 (70%)	
Stage IV	65 (67.01%)	30 (66.66%)	35 (67.30%)		34 (52.3%)	31 (47.6%)		23 (35.3%)	42 (64.6%)	
Chemotherapy				0.96			0.95			0.46
Cisplatin	72 (74.22%)	34 (75.55%)	38 (73.07%)		36 (50%)	36 (50%)		25 (34.7%)	47 (65.2%)	
Paclitaxel/Carboplatin	25 (25.77%)	11 (24.44%)	14 (26.92%)		13 (52%)	12 (48%)		6 (24%)	19 (76%)	
Dysphagia RT end (CTCAE)				0.98			0.05			0.28
1–2	34 (35.05%)	15 (33.33%)	19 (36.53%)		12 (35.2%)	22 (64.7%)		13 (38.2%)	21 (61.7%)	
3–4	63 (64.94%)	30 (66.66%)	33 (63.46%)		37 (58.7%)	26 (41.2%)		16 (25.3%)	47 (74.6%)	

**Table 2:** Univariate and multivariate Cox-regression on factors influencing overall survival.

	Univariate Analysis Hazard Ratio (95% CI)	*p*-Value	Multivariate Analysis Hazard Ratio (95% CI)	*p*-Value
Gender				
Male	Ref			
Female	1.25 (0.66–2.34)	0.49		
Age	1.01 (0.98–1.05)	0.46		
BMI	0.92 (0.87–0.98)	>0.01	0.93 (0.88–0.99)	0.02
Hb level week 1	0.91 (0.78–1.07)	0.24		
Hb level week 2	0.86 (0.74–1.00)	0.04		
Hb level week 3	0.83 (0.70–0.98)	0.03		
Hb level week 4	0.81 (0.68–0.97)	0.02		
Hb level week 5	0.80 (0.65–0.97)	0.02		
Hb level week 6	0.78 (0.65–0.94)	>0.01	0.81 (0.68–0.98)	0.04
Hb difference week 1–6	1.15 (0.95–1.41)	0.16		

## Data Availability

The original contributions presented in this study are included in the article. Further inquiries can be directed to the corresponding author.

## Abbreviations

The following abbreviations are used in this manuscript:

AJCC	American Joint Committee on Cancer
BMI	Body Mass Index
CI	Confidence Interval
CTCAE	Common Terminology Criteria for Adverse Events
EBRT	External Beam Radiotherapy
Gy	Gray
Hb	Hemoglobin
HNSCC	Head and Neck Squamous Cell Carcinoma
IMRT	Intensity-Modulated Radiotherapy
OS	Overall Survival
PFS	Progression-Free Survival
RCT	Radiochemotherapy (combined radiotherapy and chemotherapy)
RTOG	Radiation Therapy Oncology Group
SD	Standard Deviation
SPSS	Statistical Package for the Social Sciences
TNM	Tumor, Node, Metastasis classification
WHO	World Health Organization

## References

[1] Petit C., Lacas B., Pignon J., Le Q.T., Grégoire V., Grau C., Hackshaw A., Zackrisson B., Parmar M.K.B., Lee J. (2021). Chemotherapy and radiotherapy in locally advanced head and neck cancer: An individual patient data network meta-analysis. Lancet Oncol..

[2] Wendt T.G., Grabenbauer G.G., Rödel C.M., Thiel H., Aydin H., Rohloff R., Wustrow T.P.U., Ira H., Popella C., Schalhorn A. (1998). Simultaneous radiochemotherapy versus radiotherapy alone in advanced head and neck cancer: A randomized multicenter study. J. Clin. Oncol..

[3] Stadler P., Becker A., Feldmann H.J., Hänsgen G., Dunst J., Würschmidt F., Molls M. (1999). Influence of the hypoxic subvolume on the survival of patients with head and neck cancer. Int. J. Radiat. Oncol. Biol. Phys..

[4] Overgaard J., Hansen H.S., Overgaard M., Bastholt L., Berthelsen A., Specht L., Lindeløv B., Jørgensen K. (1998). A randomized double-blind phase III study of nimorazole as a hypoxic radiosensitizer of primary radiotherapy in supraglottic larynx and pharynx carcinoma. Results of the Danish Head and Neck Cancer Study (DAHANCA) Protocol 5-85. Radiother. Oncol..

[5] Bush R.S. (1986). The significance of anemia in clinical radiation therapy. Int. J. Radiat. Oncol. Biol. Phys..

[6] Baumeister P., Canis M., Reiter M. (2018). Preoperative anemia and perioperative blood transfusion in head and neck squamous cell carcinoma. PLoS ONE.

[7] Zhang X., Huang J., Tang M., Zhang Q., Deng L., Song C., Li W., Yang M., Shi H., Cong M. (2023). A comprehensive analysis of the association between anemia and systemic inflammation in older patients with cancer. Support Care Cancer.

[8] Hamai Y., Hihara J., Taomoto J., Yamakita I., Ibuki Y., Okada M. (2014). Hemoglobin Level Influences Tumor Response and Survival After Neoadjuvant Chemoradiotherapy for Esophageal Squamous Cell Carcinoma. World J. Surg..

[9] Göllnitz I., Inhestern J., Wendt T.G., Buentzel J., Esser D., Böger D., Mueller A.H., Piesold J., Schultze-Mosgau S., Eigendorff E. (2016). Role of comorbidity on outcome of head and neck cancer: A population-based study in Thuringia, Germany. Cancer Med..

[10] Schäfer U., Micke O., Müller S.B., Schüller P., Willich N. (2003). Hemoglobin as an Independent Prognostic Factor in the Radiotherapy of Head and Neck Tumors. Strahlenther. Onkol..

[11] Chino J., Annunziata C.M., Beriwal S., Bradfield L., Erickson B.A., Fields E.C., Fitch K., Harkenrider M.M., Holschneider C.H., Kamrava M. (2020). Radiation Therapy for Cervical Cancer: Executive Summary of an ASTRO Clinical Practice Guideline. Pract. Radiat. Oncol..

[12] Melo-Alvim C., Miguel-Semedo P., Paiva R.S., Lobo-Martins S., Luna-Pais H., Costa A.L., Santos A.R., Florindo A., Vasconcelos A.L., Abrunhosa-Branquinho A.N. (2020). Pretreatment hemoglobin level as a prognostic factor in patients with locally advanced head and neck squamous cell carcinoma. Rep. Pract. Oncol. Radiother..

[13] Kruser T.J., Rice S.R., Cleary K.P., Geye H.M., Tome W.A., Harari P.M., Kozak K.R. (2013). Acute Hematologic and Mucosal Toxicities in Head and Neck Cancer Patients Undergoing Chemoradiotherapy: A Comparison of 3D-CRT, IMRT, and Helical Tomotherapy. Technol. Cancer Res. Treat..

[14] Dhanapal R., Saraswathi T., Govind R.N. (2011). Cancer cachexia. J. Oral Maxillofac. Pathol..

[15] Eisbruch A., Harris J., Garden A.S., Chao C.K.S., Straube W., Harari P.M., Sanguineti G., Jones C.U., Bosch W.R., Ang K.K. (2010). Multi-Institutional Trial of Accelerated Hypofractionated Intensity-Modulated Radiation Therapy for Early-Stage Oropharyngeal Cancer (RTOG 00-22). Int. J. Radiat. Oncol. Biol. Phys..

[16] Wichmann J., Durisin M., Hermann R.M., Merten R., Christiansen H. (2021). Moderately Hypofractionated Intensity-modulated Radiotherapy With a Simultaneous Integrated Boost for Locally Advanced Head and Neck Cancer—Do Modern Techniques Fulfil Their Promise?. In Vivo.

[17] Edge S.B., American Joint Committee on Cancer (2010). AJCC Cancer Staging Manual.

[18] Lammering G., Carl U.M., Pape H., Hartmann K.A. (1999). Changes in hemoglobin concentrations in combined radio- and chemotherapy in locally advanced ORL tumors. Strahlenther. Onkol..

[19] Strojan P., Vermorken J.B., Beitler J.J., Saba N.F., Haigentz M., Bossi P., Worden F.P., Langendijk J.A., Eisbruch A., Mendenhall W.M. (2015). Cumulative cisplatin dose in concurrent chemoradiotherapy for head and neck cancer: A systematic review. Head Neck.

[20] Knight K., Wade S., Balducci L. (2004). Prevalence and outcomes of anemia in cancer: A systematic review of the literature. Am. J. Med..

[21] Birgegård G., Aapro M.S., Bokemeyer C., Dicato M., Drings P., Hornedo J., Krzakowski M., Ludwig H., Pecorelli S., Schmoll H. (2005). Cancer-Related Anemia: Pathogenesis, Prevalence and Treatment. Oncology.

[22] Maccio A., Madeddu C., Gramignano G., Mulas C., Tanca L., Cherchi M.C., Floris C., Omoto I., Barracca A., Ganz T. (2014). The role of inflammation, iron, and nutritional status in cancer-related anemia: Results of a large, prospective, observational study. Haematologica.

[23] Ravasco P., Monteiro-Grillo I., Marques Vidal P., Camilo M.E. (2005). Impact of nutrition on outcome: A prospective randomized controlled trial in patients with head and neck cancer undergoing radiotherapy. Head Neck.

[24] Lewis S.L., Brody R., Touger–decker R., Parrott J.S., Epstein J. (2014). Feeding tube use in patients with head and neck cancer. Head Neck.

[25] Kim T., Lee S., Shin S., Cho J., Kim K., Ha I. (2021). Sex-related associations among anemia, body mass index, and kidney function in Koreans. Medicine.

[26] Pai P., Chuang C., Tseng C., Tsang N., Chang K., Yen T., Liao C., Hong J., Chang J.T. (2012). Impact of Pretreatment Body Mass Index on Patients With Head-and-Neck Cancer Treated With Radiation. Int. J. Radiat. Oncol. Biol. Phys..

[27] Yang Z., Mansour J., Sun P., Wei P., Dahlstrom K.R., Zafereo M., Li G., Gross N.D. (2024). Impact of pretreatment body mass index on the survival of head and neck cancer patients. Head Neck.

[28] Fietkau R., Lewitzki V., Kuhnt T., Hölscher T., Hess C., Berger B., Wiegel T., Rödel C., Niewald M., Hermann R.M. (2013). A disease-specific enteral nutrition formula improves nutritional status and functional performance in patients with head and neck and esophageal cancer undergoing chemoradiotherapy: Results of a randomized, controlled, multicenter trial. Cancer.

[29] Chen Z., Ling J., Zhang S., Feng Y., Xie Y., Liu X., Hou T. (2024). Predicting the overall survival and progression-free survival of nasopharyngeal carcinoma patients based on hemoglobin, albumin, and globulin ratio and classical clinicopathological parameters. Head Neck.

[30] Yang M., Chen T., Wang H., Hsieh J.C., Huang H., Hsieh M., Yen C., Wu S., Hua C., Lien M. (2024). Prognostic factors and risk-stratification model of recurrent or metastatic head and neck squamous cell carcinoma treated with cetuximab containing regimen. BMC Cancer.

